# Supplementary material for: General approach to surface-accessible plasmonic Pickering emulsions for SERS sensing and interfacial catalysis
Source: Nat Commun. 2023 Mar 13;14:1392. doi: 10.1038/s41467-023-37001-1 (PMC10011407; doi:10.1038/s41467-023-37001-1)
Supplement: Supplementary file 1 — Supplementary Information [file 41467_2023_37001_MOESM1_ESM.pdf]

Supplementary information

General approach to surface-accessible plasmonic Pickering emulsions for SERS sensing and interfacial catalysis

Yingrui Zhang, Ziwei Ye, Chunchun Li, Qinglu Chen, Wafaa Aljuhani, Yiming Huang, Xin Xu, Chunfei Wu, Steven E. J. Bell, Yikai Xu*

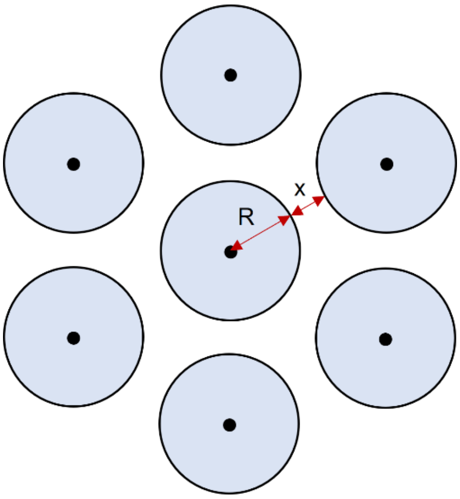

Supplementary Figure 1 | Schematic illustration of a densely packed hexagonal array of spherical nanoparticles (NPs). R corresponds to the radius of the particles, x corresponds to the surface-to-surface distance between two adjacent particles[1].

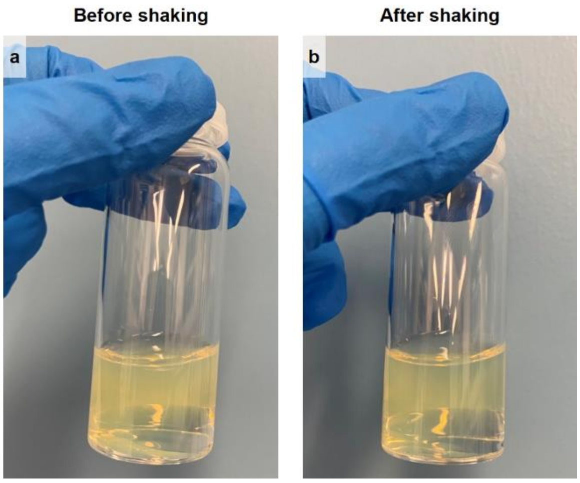

Supplementary Figure 2 | Demonstration that promoters are essential for self-assembly. Optical images showing a sample of citrate-reduced Ag nanoparticles (AgNPs) and SiO_2 nanoparticles (SiO_2NPs) before and after being shaken with dichloromethane without any promoters. The sample remains unchanged, and the NPs do not migrate to the water-oil interface. SiO_2 stabilizers were selected as an example since they are charged and dispersed in the aqueous phase.

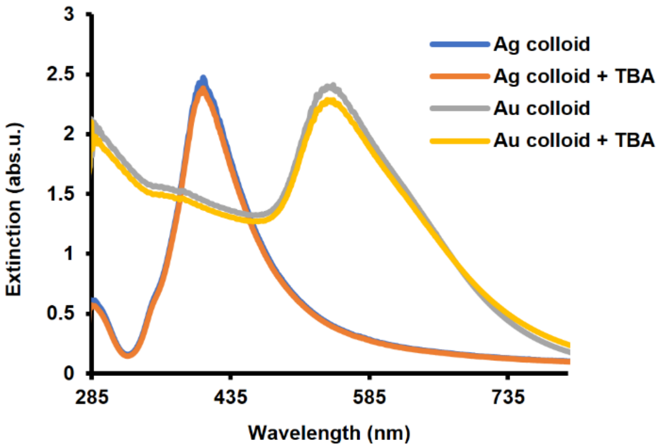

Supplementary Figure 3 | Probing the influence of tetrabutylammonium (TBA) salt on colloid stability. UV-vis spectra of citrate-reduced Ag and Au colloid with and without the addition of TBA promoters. The concentration of promoters was the same as that used for the synthesis of Pickering emulsions.

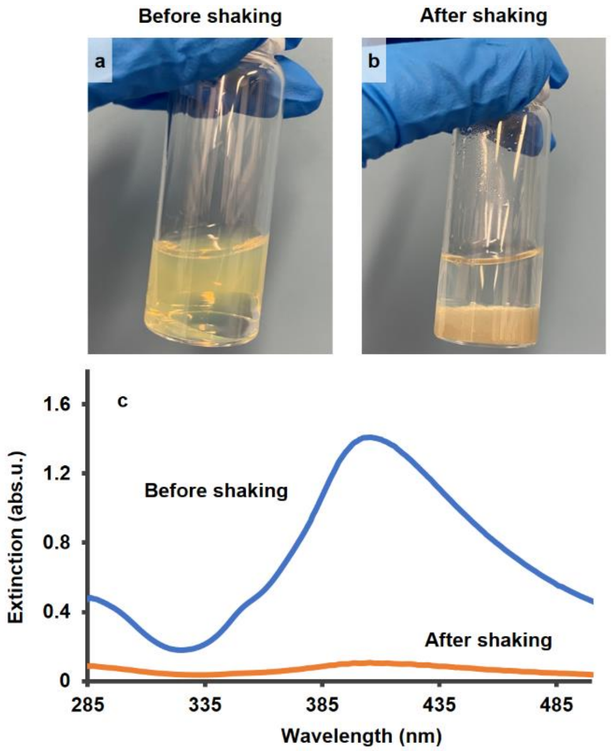

Supplementary Figure 4 | Demonstration of the migration of NPs to the water-oil interface in promoter-assisted self-assembly. (a)-(b) Optical images showing a sample of citrate-reduced AgNPs and SiO_2NPs before and after being shaken with dichloromethane and promoters. **(c)** UV-vis spectra of the aqueous colloid phase before and after self-assembly showing that a negligible number of NPs remain in the bulk aqueous phase after the formation of Pickering emulsions.

Supplementary Table 1 | The values of parameters p and z of Equation (4) (main text) for different cases. The values are most accurate for calculating the maximum capillary pressure between densely packed solid spherical NP bilayers.

Situation parameter	$\theta < 90°$ (o/w);<br>$\theta > 90°$ (w/o)	$90° \leq \theta \leq 129.3°$ (o/w);<br>$50.7° \leq \theta \leq 90°$ (w/o)
p	4.27	2.73
z	0.405	0.633

Supplementary Note 1 | Comparison of the maximum capillary pressure that could be withstood by the NP bilayers between two w/o CNT or Au nanoparticle (AuNP) emulsions.

As shown in Fig. 1d in the main text, the three-phase contact angle (θ) of the CNT and Au nanoparticle (AuNP) arrays were measured to be 113° and 68°, respectively.

The maximum capillary pressure (P_c^{max} that could be withstood by NP bilayers between two adjacent w/o Pickering emulsions can be calculated using Equation (4) in the main text, which is also shown below as Supplementary Equation (1):

$$P_c^{max} = -p\frac{2\gamma_{wo}}{R}(\cos\theta - z) \qquad (1)$$

where γ_{wo} is the surface tension between the oil and water, R is the radius of the NP, and the emulsion system becomes more stable at higher P_c^{max} values.

Since $\theta_{CNT} = 113° > 90°$ and $\theta_{Au} = 68° \geq 50.7°$, according to Supplementary Table 1, this means that:

$$P_c^{max}(\mathrm{CNT}) = -4.27 \times \frac{2\gamma_{wo}}{R_{CNT}} \times (\cos 113° - 0.405) \approx 6.8\frac{\gamma_{wo}}{R_{CNT}} \qquad (2)$$

$$P_c^{max}(\mathrm{Au}) = -2.73 \times \frac{2\gamma_{wo}}{R_{Au}} \times (\cos 68° - 0.633) \approx 1.4\frac{\gamma_{wo}}{R_{Au}} \qquad (3)$$

Since the value of R_{Au} is similar to R_{CNT}, this means that $P_c^{max}(\mathrm{CNT}) > P_c^{max}(\mathrm{Au})$, and that CNT stabilized w/o Pickering emulsions are more stable against coalescence than AuNP stabilized Pickering emulsions.

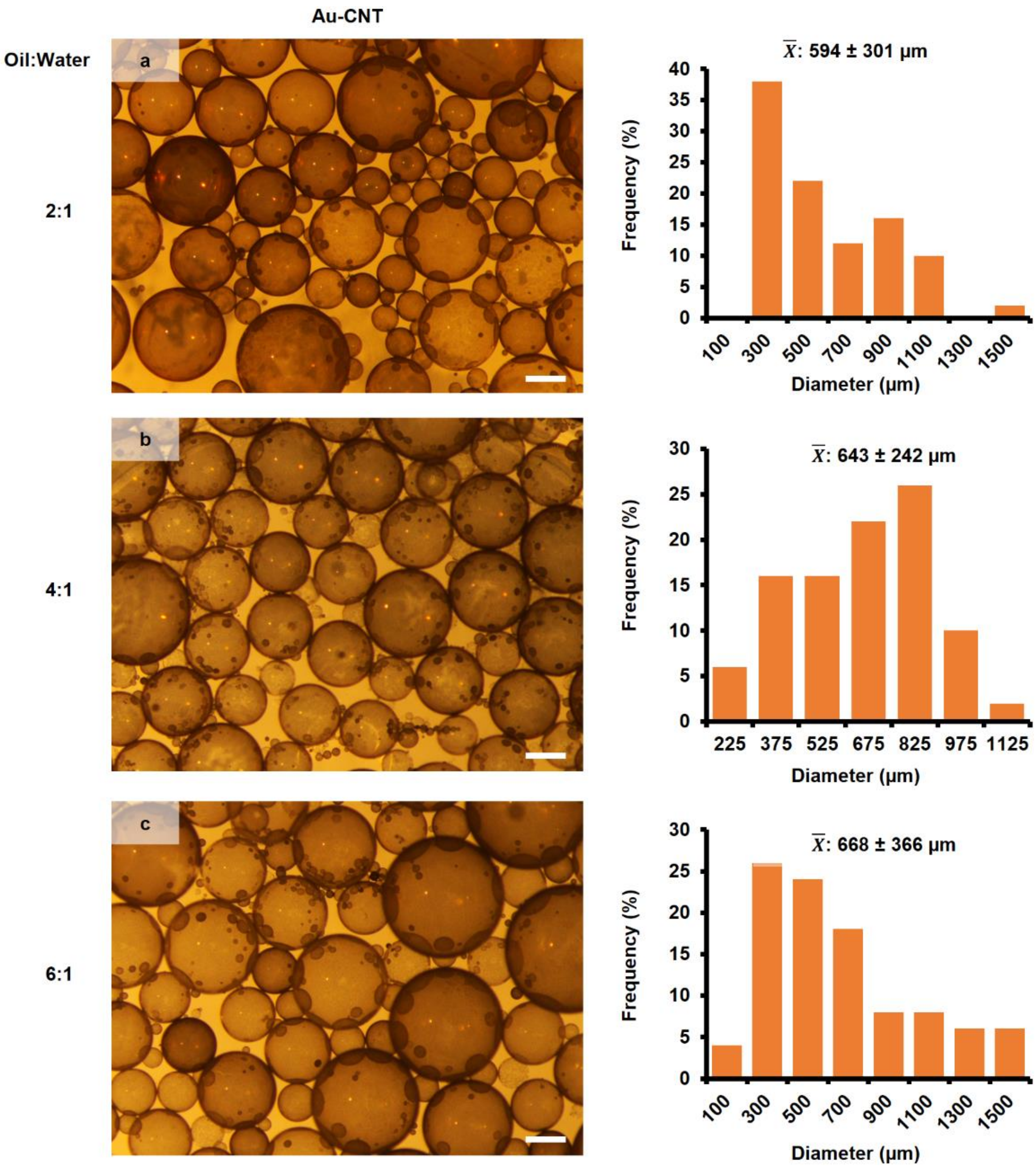

Supplementary Figure 5 | The influence of water-oil ratio on the average size of Pickering emulsions. Optical microscopy images and size distribution charts showing the average size of CNT-AuNP Pickering emulsion at different oil to water ratios. The optical image in (b) is also shown in Supplementary Fig. 7c. The scale bars correspond to 500 μm.

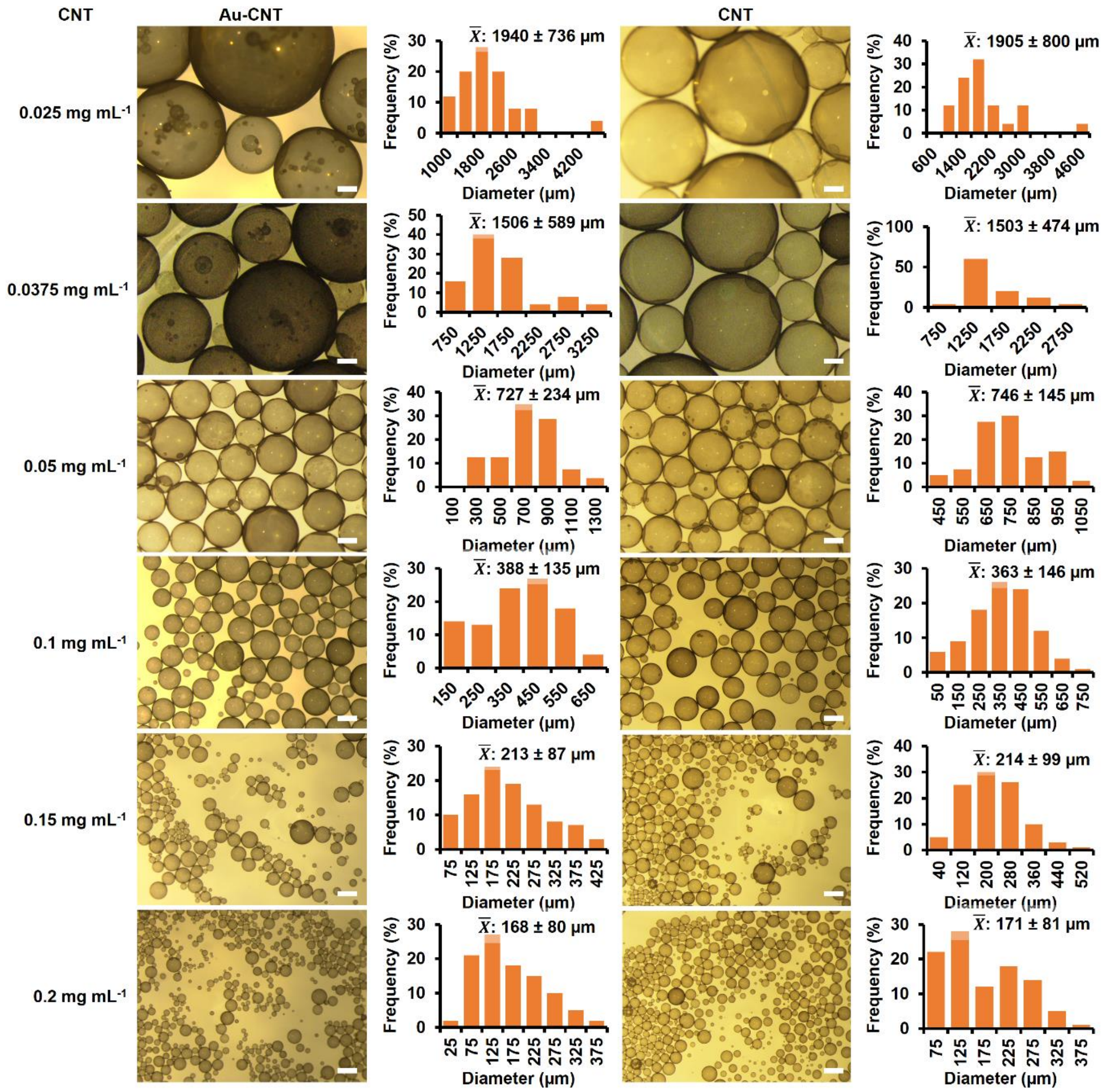

Supplementary Figure 6 | Comparison of the average size of CNT-AuNP and CNT emulsions at different CNT concentrations. Optical microscopy images and size distribution charts showing the average size of CNT-AuNP Pickering emulsions and CNT Pickering emulsions formed using different concentrations of CNTs. The scale bars correspond to 500 µm. The optical images for CNT-AuNP emulsions are also presented in Fig. 3 of the main text.

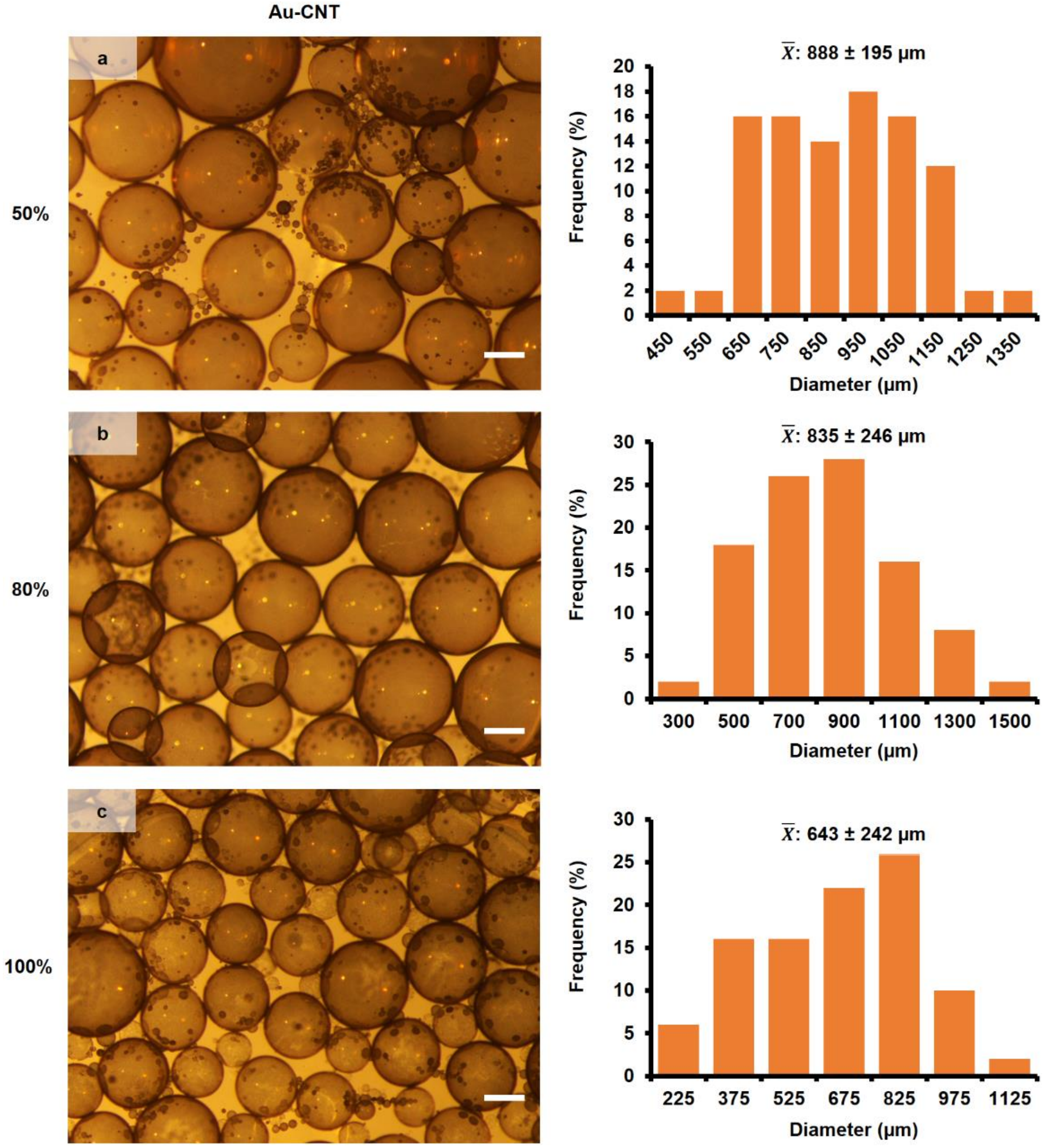

Supplementary Figure 7 | The average size of CNT-AuNP emulsions formed with different AuNP concentrations. (a)-(c) Optical microscopy images and size distribution charts showing the average size of CNT-AuNP Pickering emulsions formed using citrate-reduced Au colloid diluted by 2× (50%), 1.25× (80%) and at original concentration (100%), respectively. The optical image in (c) is also shown in Supplementary Fig. 5b. The scale bars correspond to 500 µm.

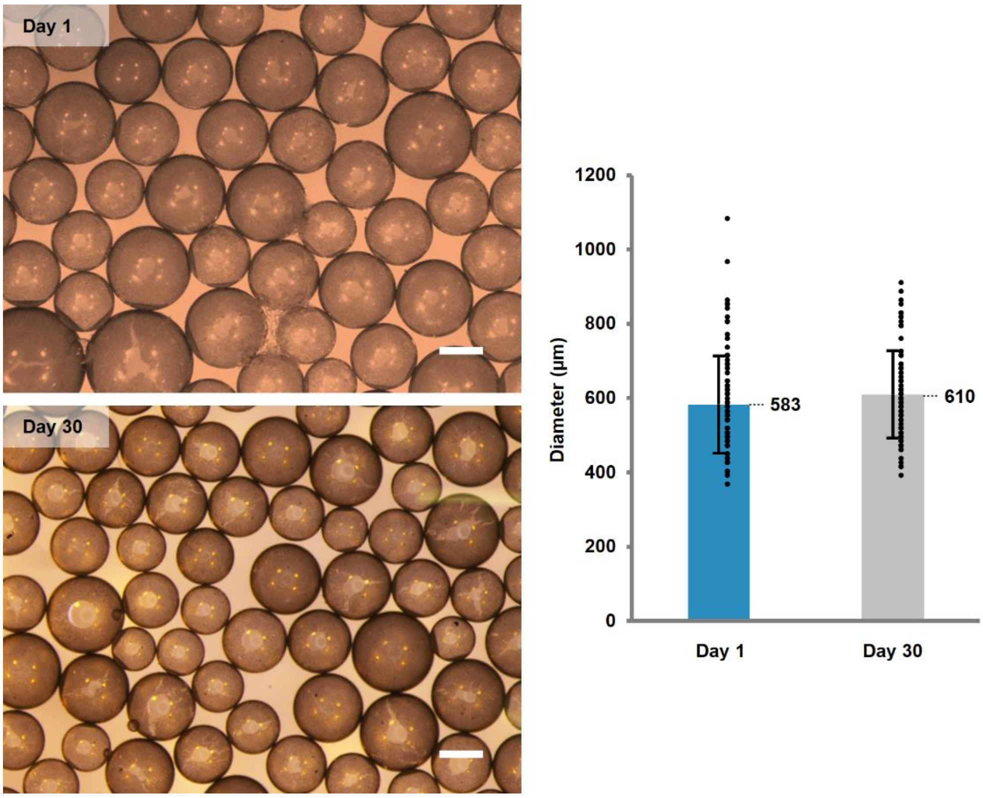

Supplementary Figure 8 | Microscopic imaging for characterization of the stability of CNT-AuNP emulsions. Optical images and the average size of a typical batch of CNT-AuNP emulsions formed with dichloromethane as the oil phase containing 0.05 mg mL^{-1} of CNT before and after being stored for one month. The scale bars in the optical images correspond to 500 µm. The error corresponding to each bar in the chart were calculated from one hundred emulsion droplets randomly selected from three independent emulsion samples.

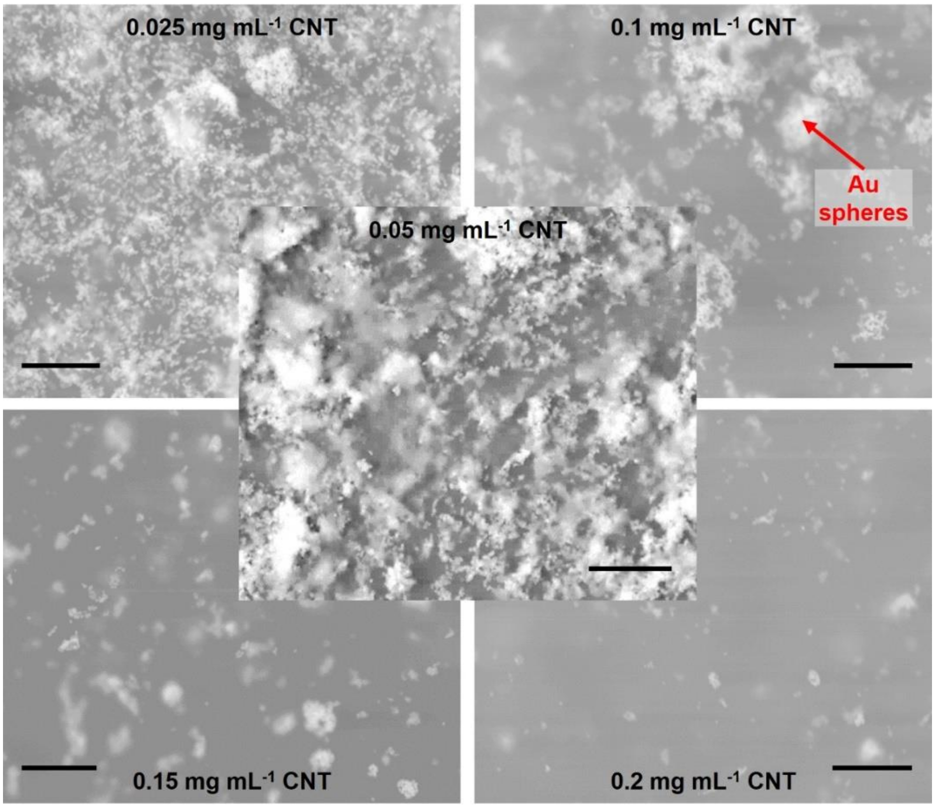

Supplementary Figure 9 | Probing the arrangement of CNT and AuNPs in CNT-AuNP emulsions. SEM images of the mixed NP layer in CNT-AuNP emulsions stabilized with different concentrations of CNTs imaged using a backscattering detector so that only the AuNPs are visible. The scale bars correspond to 1 µm.

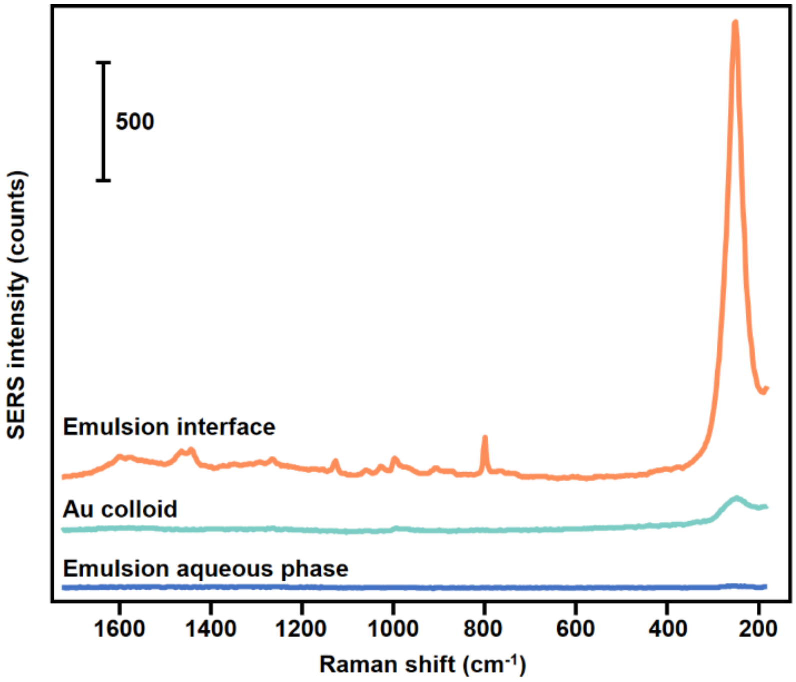

Supplementary Figure 10 | Confocal SERS microscopy showing the localization of NPs and the formation of plasmonic hot spots at the water-oil interface. SERS spectra obtained from the surface (water-oil interface) of a CNT-AuNP emulsion droplet (light orange), from the parent Au colloid (teal) and the bulk aqueous phase of the same CNT-AuNP emulsion droplet (blue).

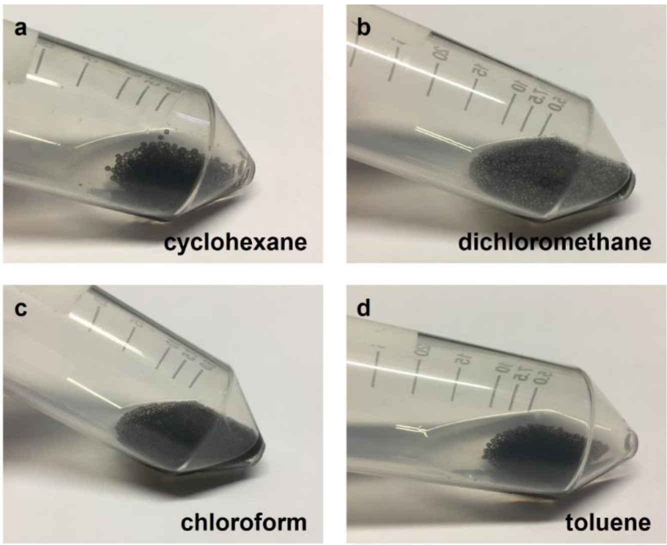

Supplementary Figure 11 | Pickering emulsions formed with different oils. Optical images of CNT-AuNP Pickering emulsions formed using **(a)** cyclohexane, **(b)** dichloromethane, **(c)** chloroform, and **(d)** toluene.

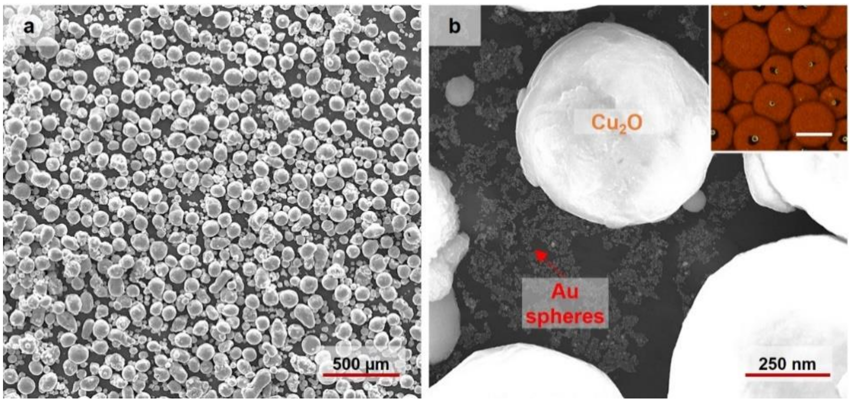

Supplementary Figure 12 | Characterizations of Cu_2O-AuNP emulsions. (a)-(b) SEM images of the mixed NP layer at different magnifications. Inset shows the optical microscopy image of a typical batch of Cu_2O-AuNP Pickering emulsions. The scale bar in the inset corresponds to 500 µm.

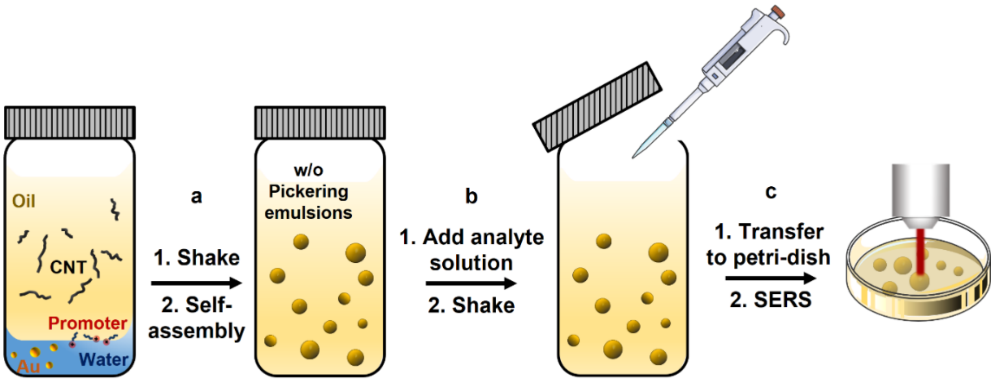

Supplementary Figure 13 | Protocol for performing SERS studies with CNT-AuNP emulsions. Schematic illustration of the experimental process for performing SERS analysis using CNT-AuNP Pickering emulsions as the enhancing substrate.

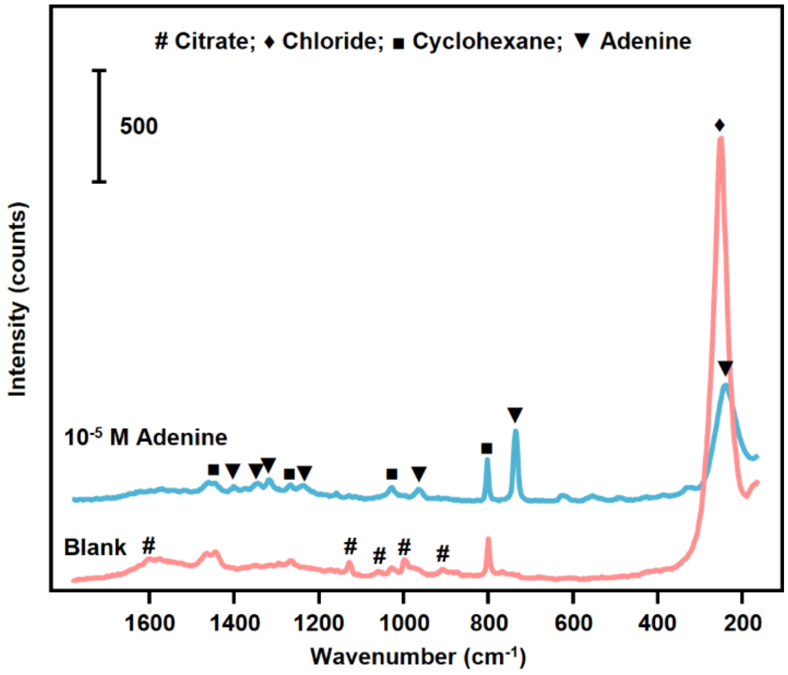

Supplementary Figure 14 | Probing the adsorption of adenine on modifier-free CNT-Au emulsions via SERS. SERS spectra obtained from CNT-AuNP emulsions before (rose) and after (turquoise) interacting with 10^{-5} M of adenine.

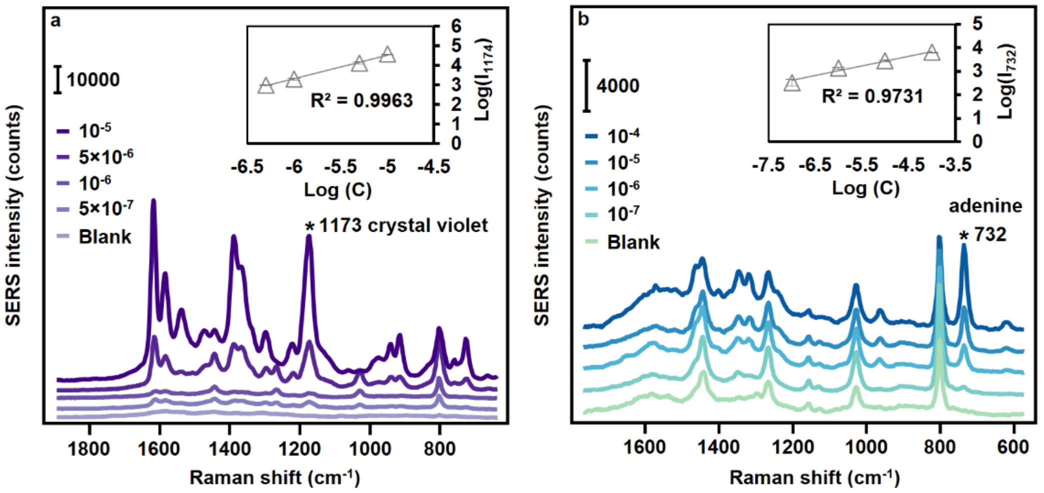

Supplementary Figure 15 | SERS Quantitation with CNT-AuNP emulsions. (a)-(b) SERS quantitation of crystal violet and adenine using CNT-AuNP emulsions as the enhancing substrate. Insets show the calibration curves obtained by plotting Log ($C_{analyte}$) versus Log($I_{analyte}$).

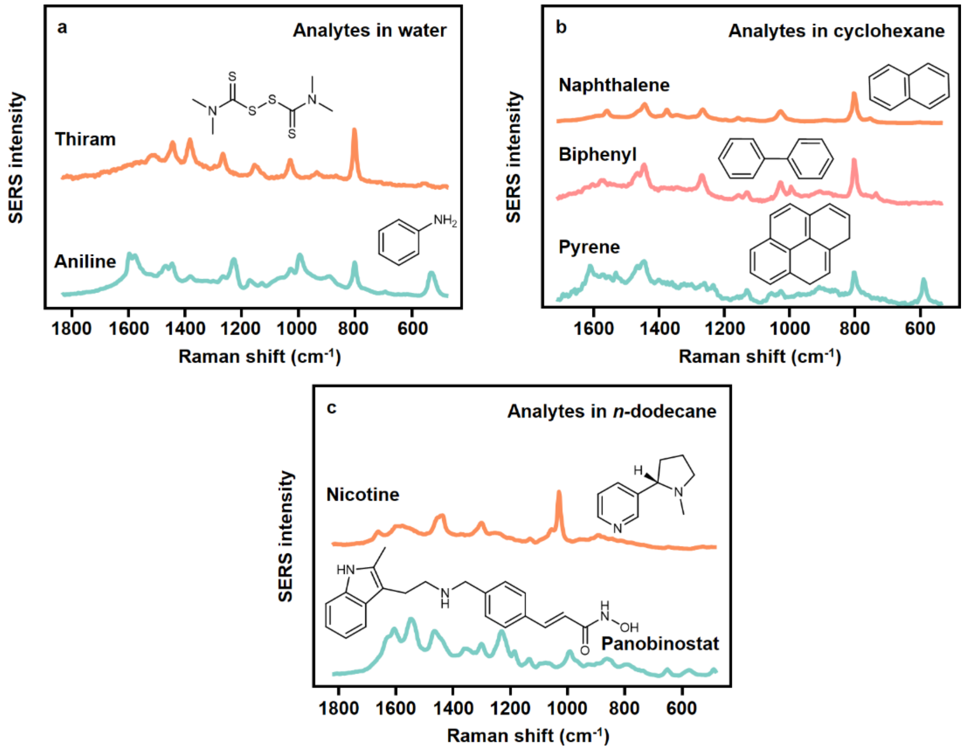

Supplementary Figure 16 | SERS of various important analytes obtained using w/o CNT-AuNP emulsions as the enhancing substrate. (a) SERS spectra of CNT-AuNP emulsions with 10^{-5} M of thiram (light orange) or 10^{-4} M of aniline (teal) introduced to the aqueous phase. **(b)** SERS spectra of CNT-AuNP emulsions with 10^{-4} M of naphthalene (light orange), biphenyl (rose), or pyrene (teal) introduced to the cyclohexane oil phase. **(c)** SERS spectra of CNT-AuNP emulsions with 10^{-4} M of nicotine (light orange) or 10^{-5} M panobinostat (teal) introduced to the *n*-dodecane oil phase. The SERS intensity of the spectra have been normalized for illustration purposes. The spectra of aniline, naphthalene and nicotine are also displayed in Fig. 5 of the main text.

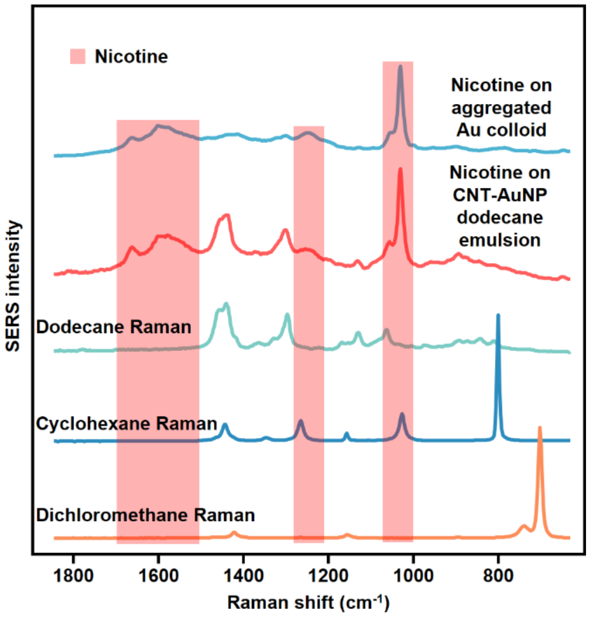

Supplementary Figure 17 | SERS performed using CNT-AuNP emulsions formed with *n*-dodecane as the oil phase. Reference SERS spectrum of nicotine obtained using aggregated citrate-reduced Au colloid (turquoise). SERS spectrum of nicotine obtained using CNT-AuNP Pickering emulsions formed with *n*-dodecane (rose). Raman spectra of *n*-dodecane (teal), cyclohexane (light blue) and dichloromethane, respectively, showing that the characteristic peaks of nicotine overlaps with cyclohexane (light orange). The SERS intensity of the spectra have been normalized for illustration purposes. The spectrum of nicotine on CNT-AuNP emulsion is also displayed in Fig. 5 of the main text.

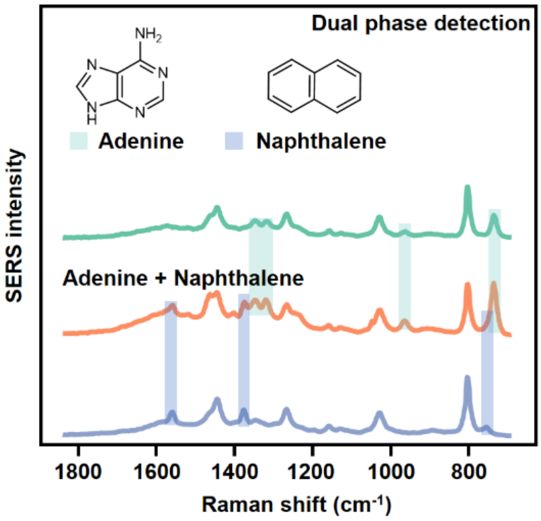

Supplementary Figure 18 | Biphasic SERS analysis performed using CNT-AuNP emulsions. SERS spectra of CNT-AuNP emulsions with 10^{-6} M of adenine introduced to the aqueous phase (teal) or 10^{-4} M of naphthalene to the oil phase (light blue), or 10^{-6} M of adenine and 10^{-4} M of naphthalene introduced simultaneously to the aqueous and oil phases (light orange). The SERS intensity of the spectra have been normalized for illustration purposes. Spectrum ii is also displayed in Fig. 5c of the main text.

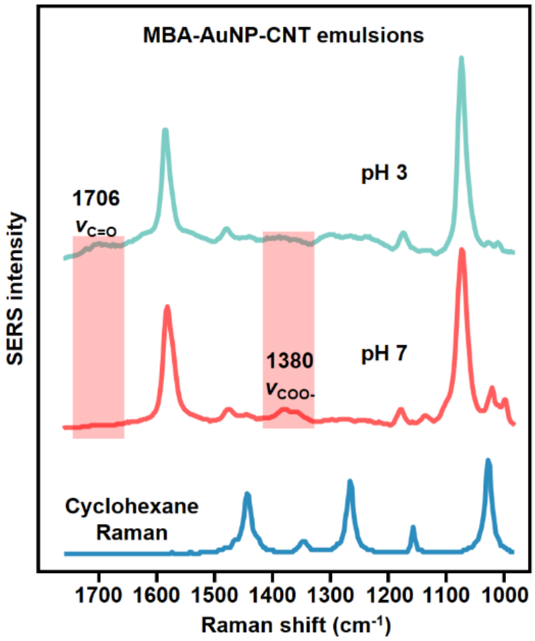

Supplementary Figure 19 | Demonstrating the potential of surface-functionalized CNT-AuNP emulsions as SERS pH sensors. SERS of 4-mercaptobeozoic acid (MBA) at different pH values obtained using CNT-AuNP Pickering emulsions. The SERS intensity of the spectra have been normalized for illustration purposes.

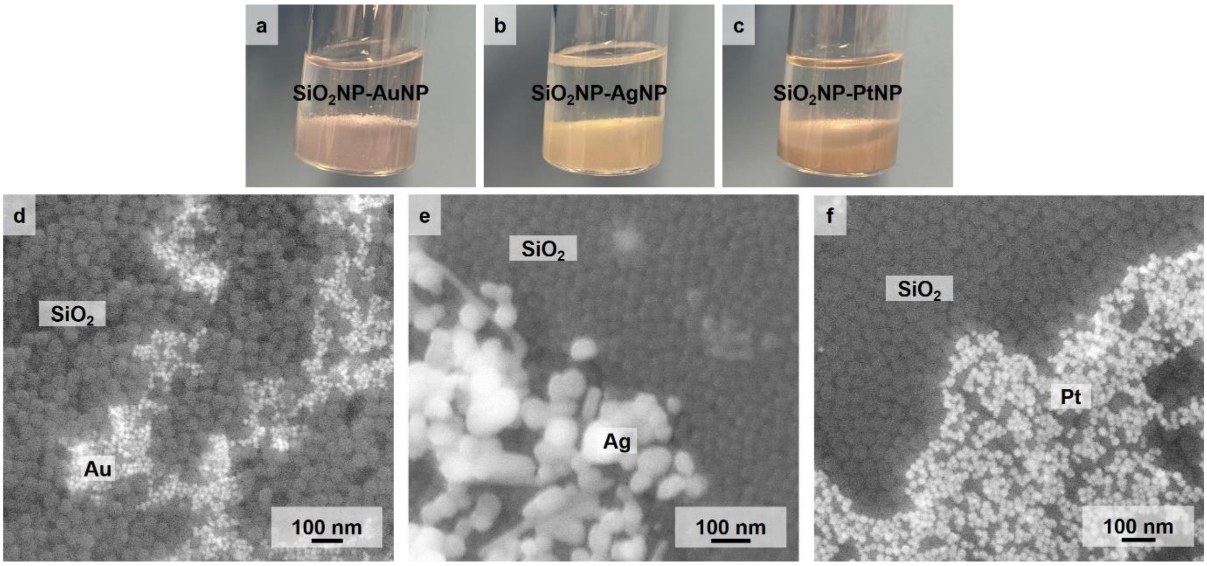

Supplementary Figure 20 | SiO_2NP-metal NP emulsions for catalysis. (a)-(c) Optical images of typical batches of SiO_2NP-AuNP, AgNP and PtNP Pickering emulsions, respectively. **(d)-(f)** SEM images of SiO_2NP-AuNP, AgNP and PtNP Pickering emulsions.

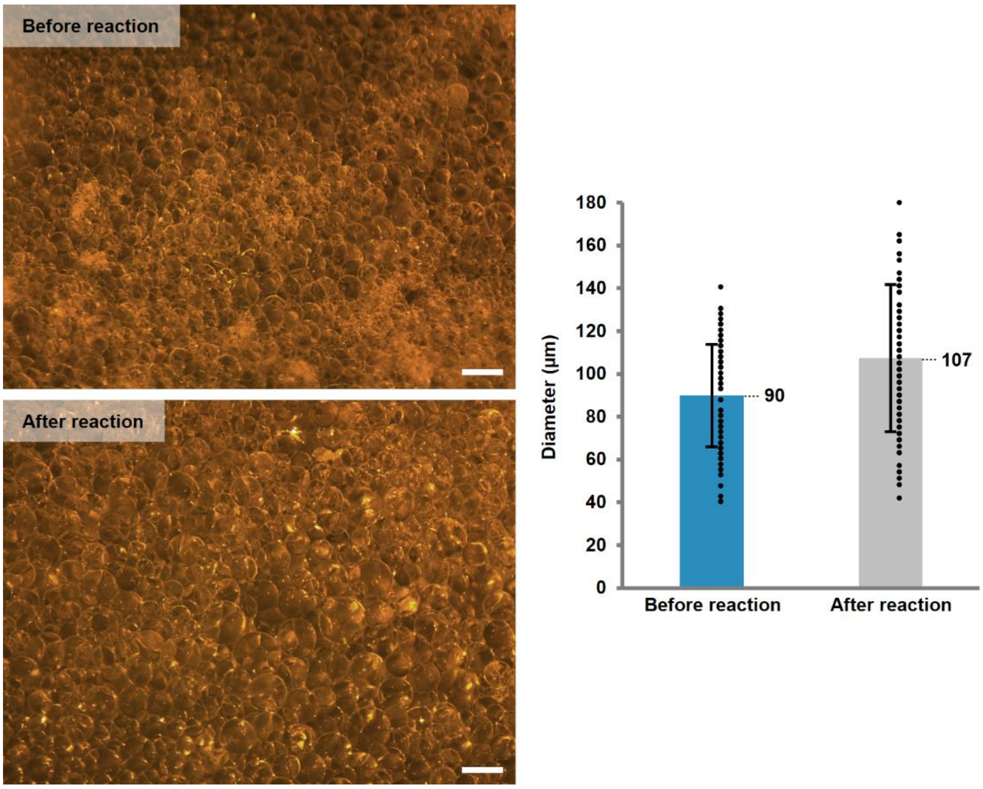

Supplementary Figure 21 | Probing the stability of SiO_2NP-AgNP emulsions after acting as interfacial catalysts. Optical microscopy images of a typical batch of SiO_2NP-AgNP emulsion before and after catalytic reduction of 4-nitrophenol. The plot shows the average diameter and standard deviation of the emulsion droplets measured from the two images. The scale bars in the optical images correspond to 500 µm. The error corresponding to each bar in the chart were calculated from one hundred emulsion droplets randomly selected from one emulsion sample.

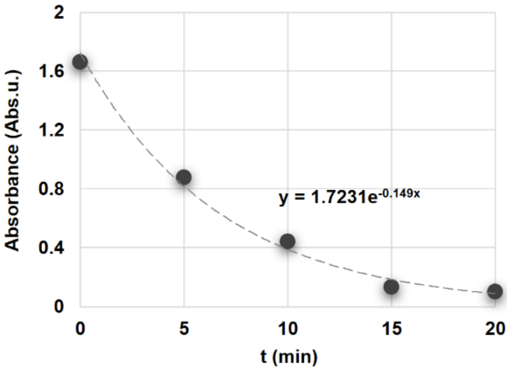

Supplementary Figure 22 | Determination of the reaction order of 4-nitrophenol catalytic reduction by SiO_2-Ag emulsions. The signal intensity of 4-nitrophenol measured with UV-vis spectroscopy at different points of its catalytic reduction plotted against reaction time.

Supplementary Note 2 | Calculation of reaction rate and conversion rate.

The signal intensity of 4-nitrophenol is directly proportional to the concentration of 4-nitrophenol (Beer-Lambert law). As shown in the data above, the signal intensity of 4-nitrophenol plotted versus time can be fitted with an exponential function which shows that it is a pseudo first order rate reaction. Therefore, the reaction rate constant, k, can be calculated following the equation:

$$Ln\frac{A}{A_0} = -kt \qquad (4)$$

Plotting $Ln\frac{A}{A_0}$ against t, as shown in Fig. 6e of the main text, gives the value of k_{Ag}, which is 0.1548 min^{-1}.

From Supplementary Fig. S22, it can be seen that the reaction is finished after ca. 20 minutes. The overall conversion can be calculated following the equation:

$$(1-\frac{A_{20}}{A_0})\times 100\% \qquad (5)$$

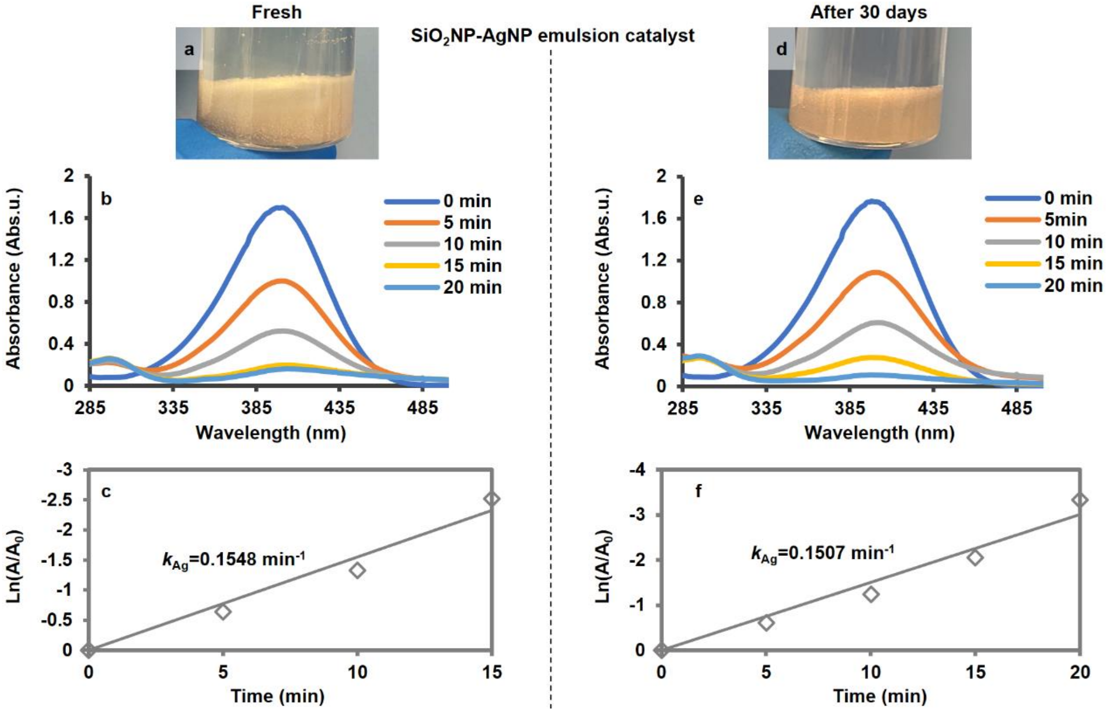

Supplementary Figure 23 | Comparing the catalytic activity of fresh and stored SiO_2-Ag emulsions. (a) Optical image of a typical batch of freshly prepared SiO_2NP-AgNP emulsions. **(b)-(c)** UV-vis spectra of 4-nitrophenol measured at different stages of the catalytic reduction using freshly prepared SiO_2NP-AgNP emulsions as the catalyst, and the Ln(A/A_0) of 4-nitrophenol measured by UV-vis spectroscopy plotted against time. **(d)** Optical image of a typical batch of SiO_2NP-AgNP emulsions that had been stored at room temperature for 30 days. **(e)-(f)** UV-vis spectra of 4-nitrophenol measured at different stages of the catalytic reduction using SiO_2NP-AgNP emulsions that had been stored for 30 days as the catalyst, and the Ln(A/A_0) of 4-nitrophenol measured by UV-vis spectroscopy plotted against time. The data in panels (b) and (c) are also displayed in Fig. 6 of the main text. Panel (b) is also displayed in Supplementary Fig. 24-27.

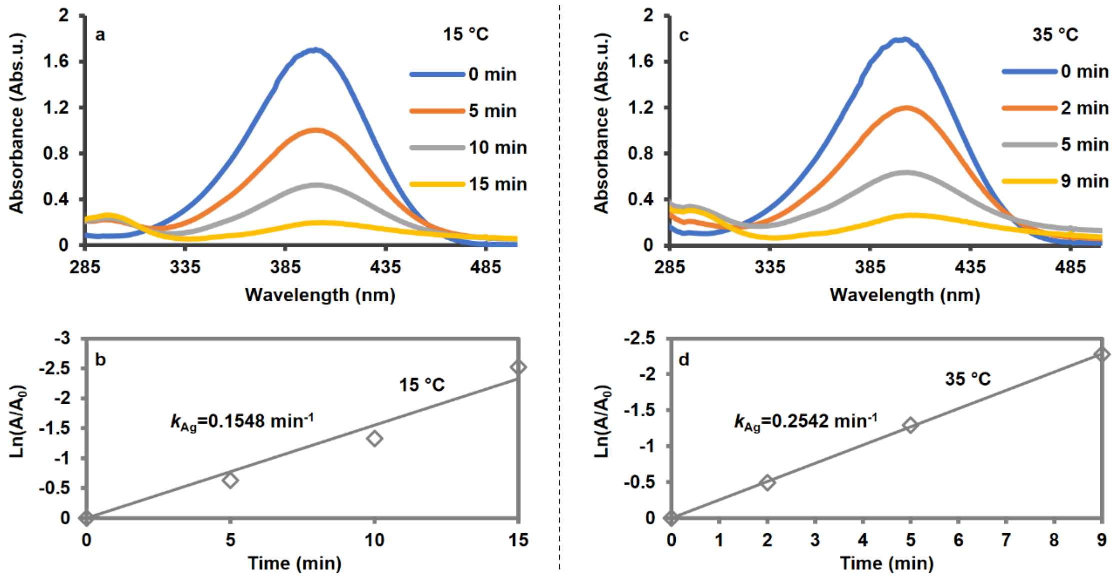

Supplementary Figure 24 | Effect of temperature on the catalytic performance of SiO_2NP-AgNP emulsions. (a)-(d) UV-vis spectra of 4-nitrophenol measured at different stages of the catalytic reduction using SiO_2NP-AgNP emulsions as the catalyst at 15 °C and 35 °C, respectively, and the Ln(A/A_0) of 4-nitrophenol measured by UV-vis spectroscopy plotted against time. Panels (a) and (b) are also displayed in Fig. 6 of the main text. Panel (a) is also displayed in Supplementary Fig. 23, 25, 26 and 27.

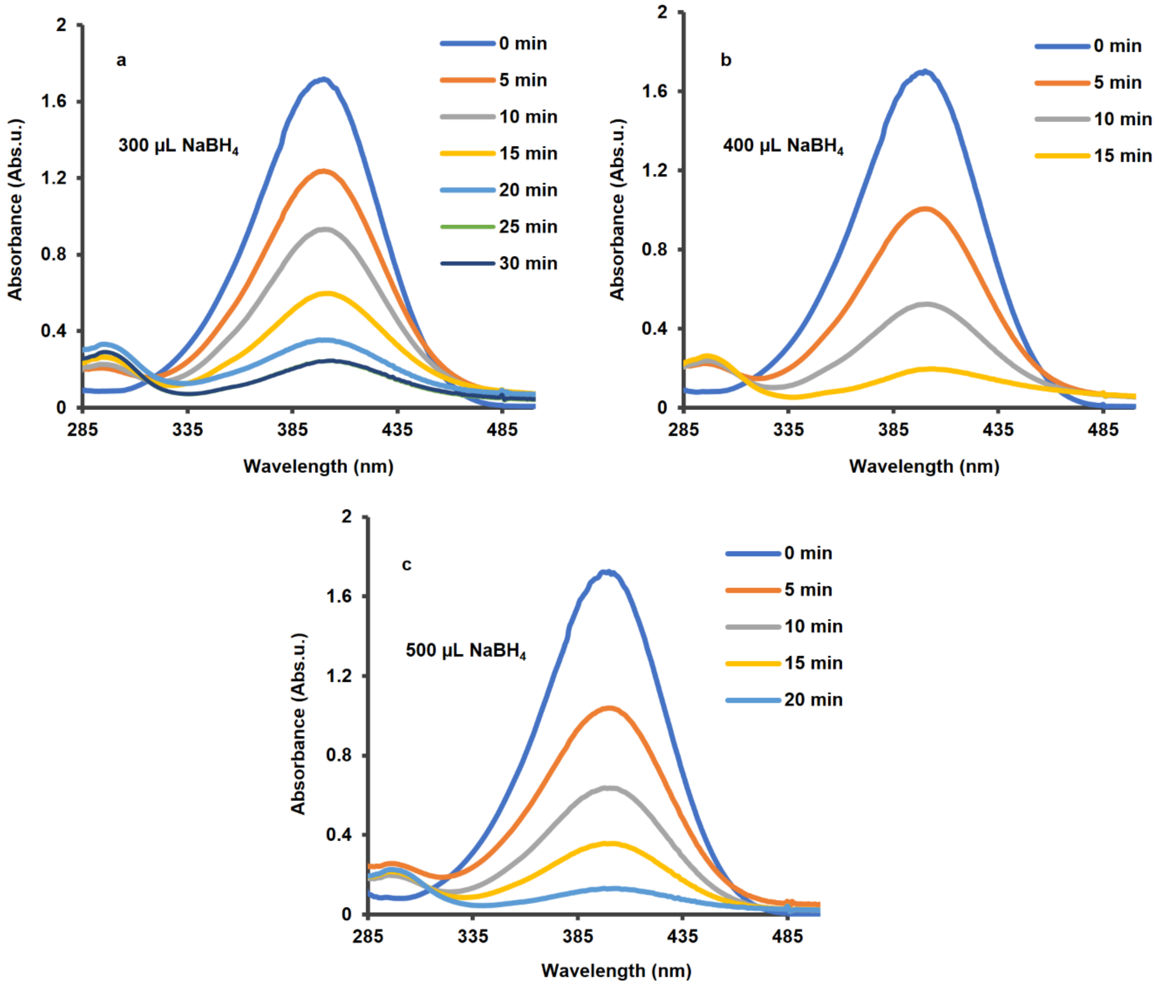

Supplementary Figure 25 | Effect of reactant concentrations on the catalytic performance of SiO_2NP-AgNP emulsions. (a)-(c) UV-vis spectra of 4-nitrophenol measured at different stages of the catalytic reduction using SiO_2NP-AgNP Pickering emulsions as the catalyst at various $NaBH_4$ concentrations. Panel (b) is also displayed in Fig. 6 of the main text and Supplementary Fig. 23, 24, 26 and 27.

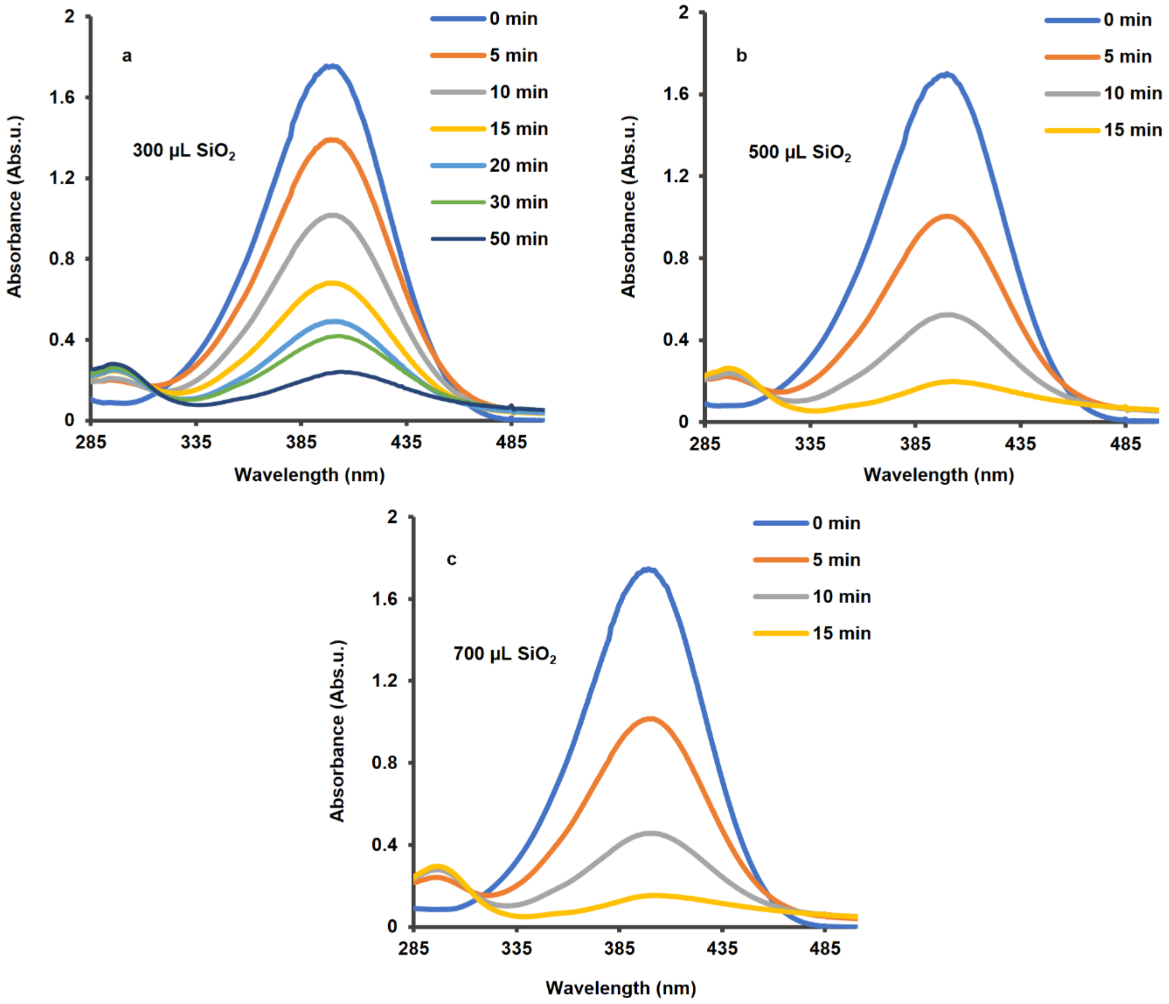

Supplementary Figure 26 | Effect of stabilizer concentrations on the catalytic performance of SiO_2-Ag emulsions. (a)-(c) UV-vis spectra of 4-nitrophenol measured at different stages of the catalytic reduction using SiO_2-Ag Pickering emulsions as the catalyst at various SiO_2 concentrations. Panel (b) is also displayed in Fig. 6 of the main text and Supplementary Fig. 23, 24, 25 and 27.

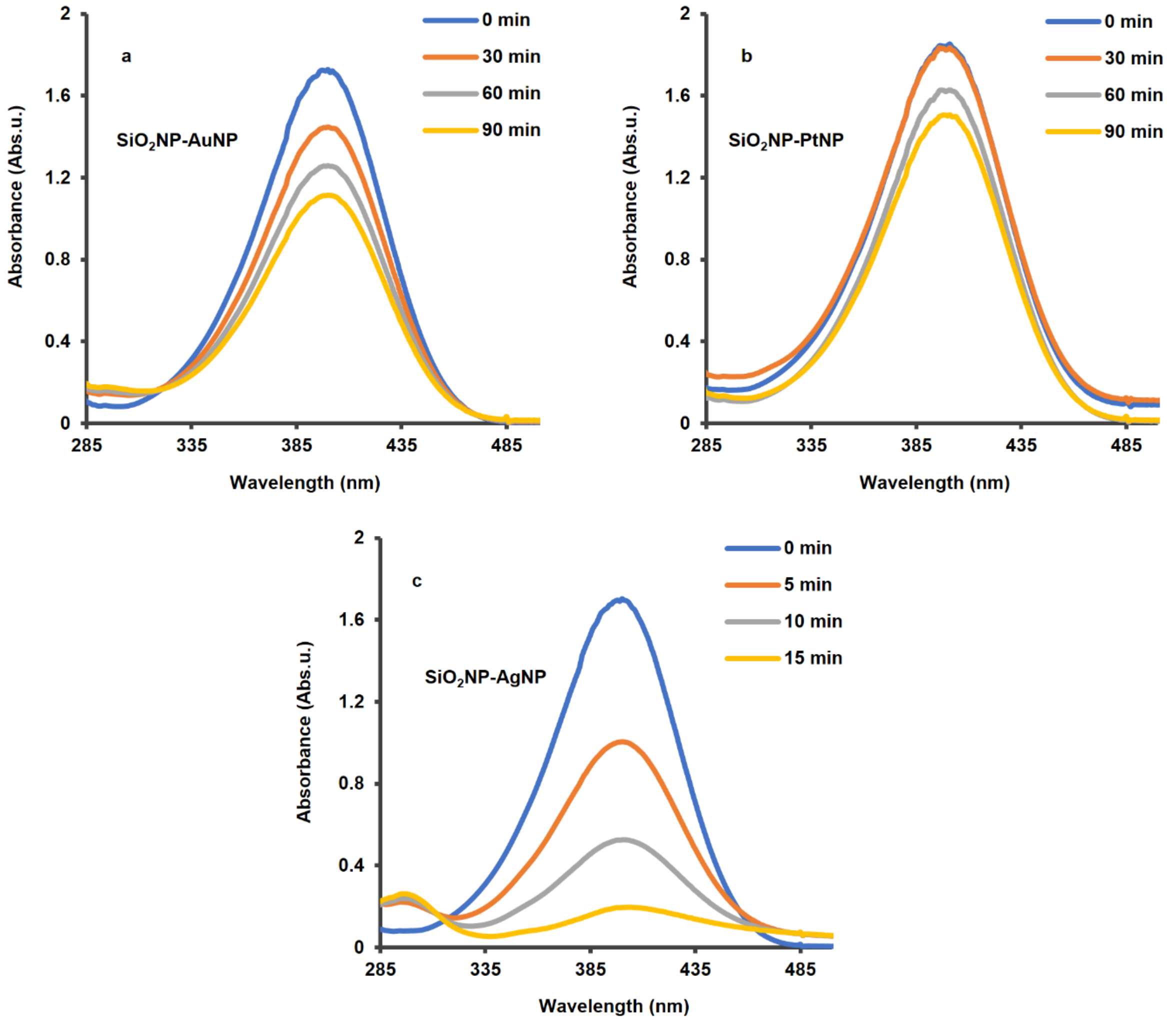

Supplementary Figure 27 | Effect of catalyst composition on the catalytic performance of SiO_2NP-metal NP emulsions. (a)-(c) UV-vis spectra of 4-nitrophenol measured at different stages of the catalytic reduction using SiO_2NP-metal NP Pickering emulsions containing different types of metal NPs. The metal catalysts weight content for AuNPs and PtNPs were 1.55×10^{-3} and 2.22×10^{-3} (wt.%), respectively. Panel (c) is also displayed in Fig. 6 of the main text and Supplementary Fig. 23, 24, 25 and 26.

Supplementary references

1. F. H. Reincke, PhD thesis, *University of Utrecht* (2004).
